# The effect of a variable focal spot size on the contrast channels retrieved in edge-illumination X-ray phase contrast imaging

**DOI:** 10.1038/s41598-022-07376-0

**Published:** 2022-03-01

**Authors:** A. Astolfo, I. Buchanan, T. Partridge, G. K. Kallon, C. K. Hagen, P. R. T. Munro, M. Endrizzi, D. Bate, A. Olivo

**Affiliations:** 1 Nikon X-Tek Systems Ltd, Tring, Herts HP23 4JX UK; 2 Department of Medical Physics and Biomedical Engineering, UCL, London, WC1E 6BT UK

**Keywords:** Engineering, Applied optics, Applied physics, Optical physics, Techniques and instrumentation

## Abstract

Multi-modal X-ray imaging allows the extraction of phase and dark-field (or “Ultra-small Angle Scatter”) images alongside conventional attenuation ones. Recently, scan-based systems using conventional sources that can simultaneously output the above three images on relatively large-size objects have been developed by various groups. One limitation is the need for some degree of spatial coherence, achieved either through the use of microfocal sources, or by placing an absorption grating in front of an extended source. Both these solutions limit the amount of flux available for imaging, with the latter also leading to a more complex setup with additional alignment requirements. Edge-illumination partly overcomes this as it was proven to work with focal spots of up to 100 micron. While high-flux, 100 micron focal spot sources do exist, their comparatively large footprint and high cost can be obstacles to widespread translation. A simple solution consists in placing a single slit in front of a large focal spot source. We used a tunable slit to study the system performance at various effective focal spot sizes, by extracting transmission, phase and dark-field images of the same specimens for a range of slit widths. We show that consistent, repeatable results are obtained for varying X-ray statistics and effective focal spot sizes. As the slit width is increased, the expected reduction in the raw differential phase peaks is observed, compensated for in the retrieval process by a broadened sensitivity function. This leads to the same values being correctly retrieved, but with a slightly larger error bar i.e. a reduction in phase sensitivity. Concurrently, a slight increase in the dark-field signal is also observed.

## Introduction

X-ray imaging is used in medicine, security, industrial testing, as well as in scientific disciplines ranging from biology to materials science. Historically, attenuation has been the main source of contrast, with different materials being distinguishable if they present a sufficiently different attenuation to the X-rays. This is reliable approach, but it suffers when the targeted details have attenuation coefficients similar to the background and/or are too thin to generate a sufficient difference in attenuation. In the above cases, phase-based X-ray imaging can provide a solution^1–3^, by exploiting the phase changes that X-rays undergo when traversing different materials to generate image contrast. This can generate stronger contrast than conventional, attenuation-based X-ray imaging in many applications.

**Figure 1:**
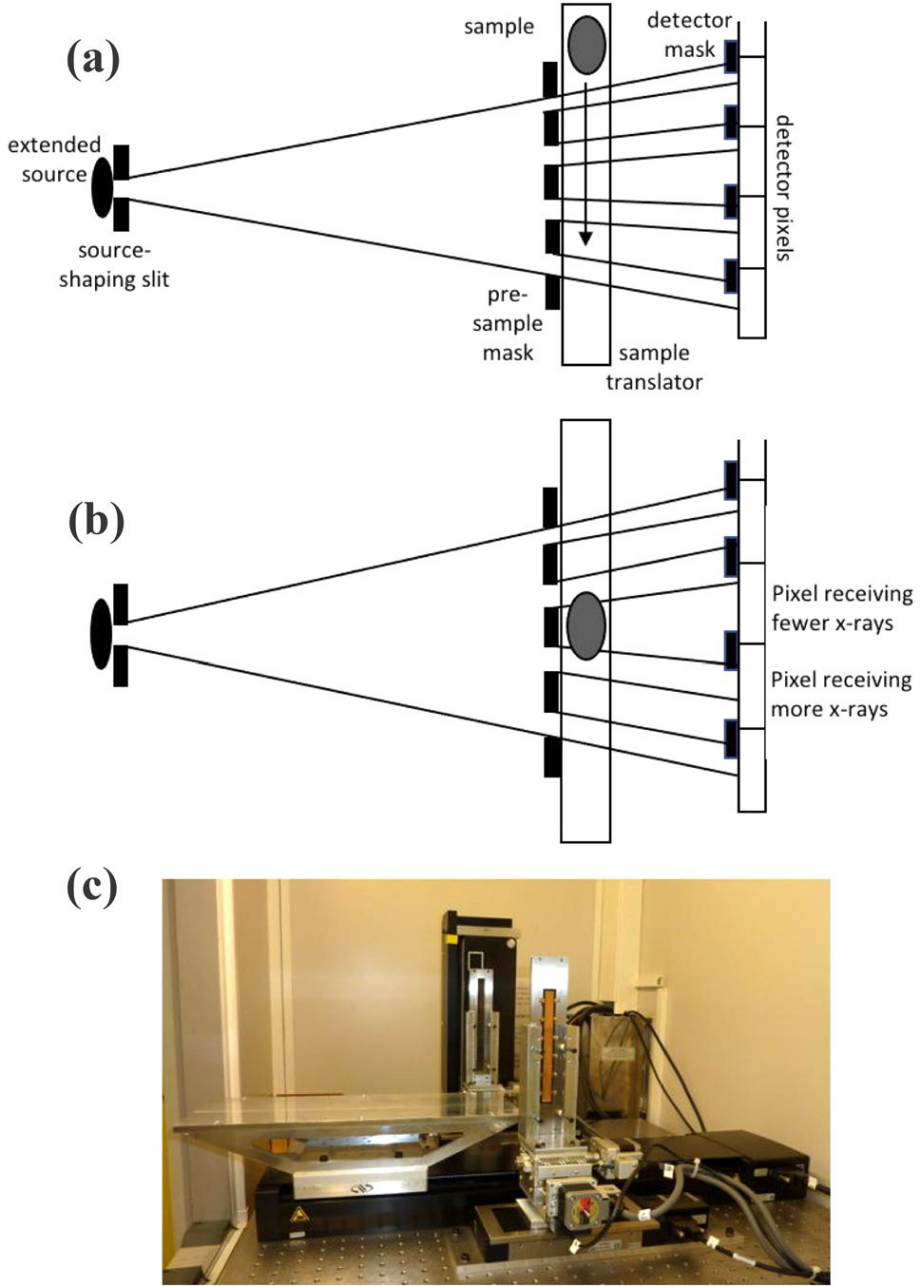
Experimental setup and edge-illumination principle. Panel (**a**) shows a top-view schematic of the setup (not to scale), with (from left to right) source, source-shaping slit, pre-sample mask, sample translation stage with sample outside the beam, detector mask, detector. Panel (**b**) shows the same with the sample at the centre of the shaped beam. As can be seen, refraction occurring at the edges of the sample (or equivalently at the edges of any detail inside the sample) pushes the relative beamlets outwards, leading to a decrease and to an increase in the number of X-rays detected by the corresponding pixels. Finally panel (**c**) shows a photo of the setup as seen from the source (not visible), with the two masks, the sample stage between them and the detector at the far end clearly visible. In this case, since we have developed a scanning-based system, masks and detector that are much longer in the vertical than in the horizontal direction have been used; however, the principle is perfectly applicable to a static system with square masks, and indeed several such systems have been built in the past.

While this is inherently an interference-based phenomenon which can be thoroughly described through wave-optics^4,5^, in the conditions of limited coherence encountered with laboratory sources, a ray-tracing approach is sufficient to provide a satisfactory description^6,7^. This corresponds to focusing on X-ray refraction: the refraction angle is proportional to the first derivative of the phase shift imprinted by an object on an X-ray wavefront^6,7^. Indeed the “edge-illumination” (EI) principle^8,9^ employed in this work is easily explained in terms of refraction (see Fig. 1).

EI is based on structuring the beam with an absorbing (“pre-sample”) mask before it hits the sample. The beam is split into multiple beamlets, one per detector pixel. A second (“detector”) mask is placed near the detector to create insensitive regions between adjacent pixels. The two masks are slightly offset, so that beamlets hit aperture edges on the detector mask. Without a sample, every pixel receives the same amount of radiation, and a “flat field” is recorded (Fig. 1a). The introduction of a sample causes the beamlets to refract, leading to a change in the number of X-rays detected by the corresponding pixels (Fig. 1b). While making the system sensitive to phase effects, this does not make it insensitive to attenuation, hence the acquired images contain a mixture of the two signals. Disentangling them (“phase retrieval”) requires imaging an object at least twice, with the pre-sample mask placed in different positions. This is typically done by acquiring the first image in the arrangement shown in Fig. 1a, and the second one after displacing the pre-sample mask so that the beamlets straddle the opposite side of the detector apertures (i.e. moving the pre-sample mask downwards by one aperture in Fig. 1a). This “inverts” the refraction-induced signal (beamlets deviated upwards cause an increase rather than decrease in the counts, and vice-versa), while leaving attenuation unchanged: simple algorithms can then be used to separate the two effects^10^. A more flexible approach, which allows phase retrieval with any number of images (≥ 2) acquired with any displacement of the pre-sample mask, is based on the mathematical inversion of the system’s “illumination curve” (IC)^11^. This is obtained by scanning the pre-sample mask (vertically in relation to Fig. 1a) while recording the detector readings: it is a bell-shaped curve with a maximum at the position where the apertures in the two masks overlap, and a minimum where they are fully misaligned. Mathematically it can be described as a convolution between projected focal spot and mask apertures^12^. The IC links the detected intensity to the beam displacement, and therefore to the refraction angle through knowledge of the distance between the masks^11^.

So far, we have considered refraction caused by details with dimensions of the order of, or larger than, the system’s spatial resolution. However, refraction by smaller features also leads to measurable contrast via a broadening of the beamlets (and therefore of the IC). This effect, referred to as “dark-field” or “Ultra-Small Angle X-Ray Scatter” imaging (“scatter” in the following), was initially observed using methods based on crystals^13–15^, then translated to grating-based methods^16^ and to EI^12^ and other approaches (e.g.^17^). Scatter imaging provides a signal related to the sample’s degree of inhomogeneity on the sub-resolution scale, which was proven to be complementary to attenuation and refraction^18^, and to be useful in several applications, including the imaging of lungs^19,20^, breast calcifications^12,21^, damage in composite materials^22^, aluminium welds^23^. To retrieve a scatter image alongside refraction and attenuation, at least three input images are required^12^; a third one with aligned mask apertures is typically added to the two “opposite partial illumination” ones used to retrieve attenuation and refraction (see above).

The need to acquire three separate images while displacing the pre-sample mask lengthens the acquisition procedures. However, in scanned acquisitions where the sample is translated across the beam (as in Fig. 1), this can be avoided by altering the pitch in the pre-sample mask, so that every aperture in the detector mask is illuminated in a different way. For example a “3-way” asymmetric pattern can be created, illuminating subsequent apertures in the detector mask on the left hand side, centre and right hand side, with this pattern repeated over the field of view^24^. Separate images are then created by combining “left-”, “centre-” and “right-illuminated” columns, which are fed to the phase retrieval algorithm to obtain attenuation, refraction and scatter images. Optionally, the retrieval process can be made more robust by introducing additional illumination points, e.g. by creating “4-way” or “5-way” asymmetric pre-sample masks^24^.

We previously used asymmetric masks to scan objects of up to 18 × 50 cm^2^ in size^25^, with the size limitation dictated by the chosen pre-sample mask and sample translation stage, both not representing a hard limit. A different approach, followed by other groups^26–28^, consists of altering the distance between the two masks or gratings along the optical axis, so that their periods are mismatched and moiré fringes are produced. Moiré fringes are equally effective in guaranteeing that different detector columns (or rows) are differently illuminated; however, illumination levels are evenly distributed, while asymmetric masks are designed specifically to target certain illumination levels (e.g. avoiding the lowest ones that cause flux and/or dose inefficiency). However, an advantage of the moiré approach is that practically no alignment is required between the two masks/gratings.

In previous systems, we used focal spots of approximately 80 μm^24,25^. While sources with focal spots around that size delivering high fluxes do exist, they tend to have larger footprints and be more expensive than average, which can limit the translation of multi-modal X-ray imaging to specialised, high-end areas. Here we utilised a conventional, large focal spot source with a tuneable slit placed in front of its exit window. As well as providing results compatible to those obtained with previous systems based on more specialised sources, this system provided the first opportunity to study experimentally the effect that a varying focal spot has on the various contrast channels. We characterised the performance of this system by extracting transmission (= 1-attenuation), phase and scatter images of a range of specimens while varying the effective focal spot size. This casts light on the effect that the focal spot has on the contrast channels; where unexpected results were observed, these have been backed up through experimentally validated simulation software. Aside from these, remarkably consistent values were retrieved over time while varying X-ray statistics and focal spot sizes.

It should be noted that we limited our exploration to the 40–100 μm focal spot range because, on the one hand, smaller focal spots were previously explored for microscopy applications^29^, but were not considered suitable for industrial inspections due to their low emitted flux. On the other hand, previous theoretical^30^ and experimental^31^ work indicated little scope for exploring focal spots larger than 100 μm due to reduced phase sensitivity. However, this work provides indications that larger focal spots could still be useful at least as far as retrieval of the scatter channel is concerned, which will be the subject of future investigations.

## Results and discussion

**Figure 2:**
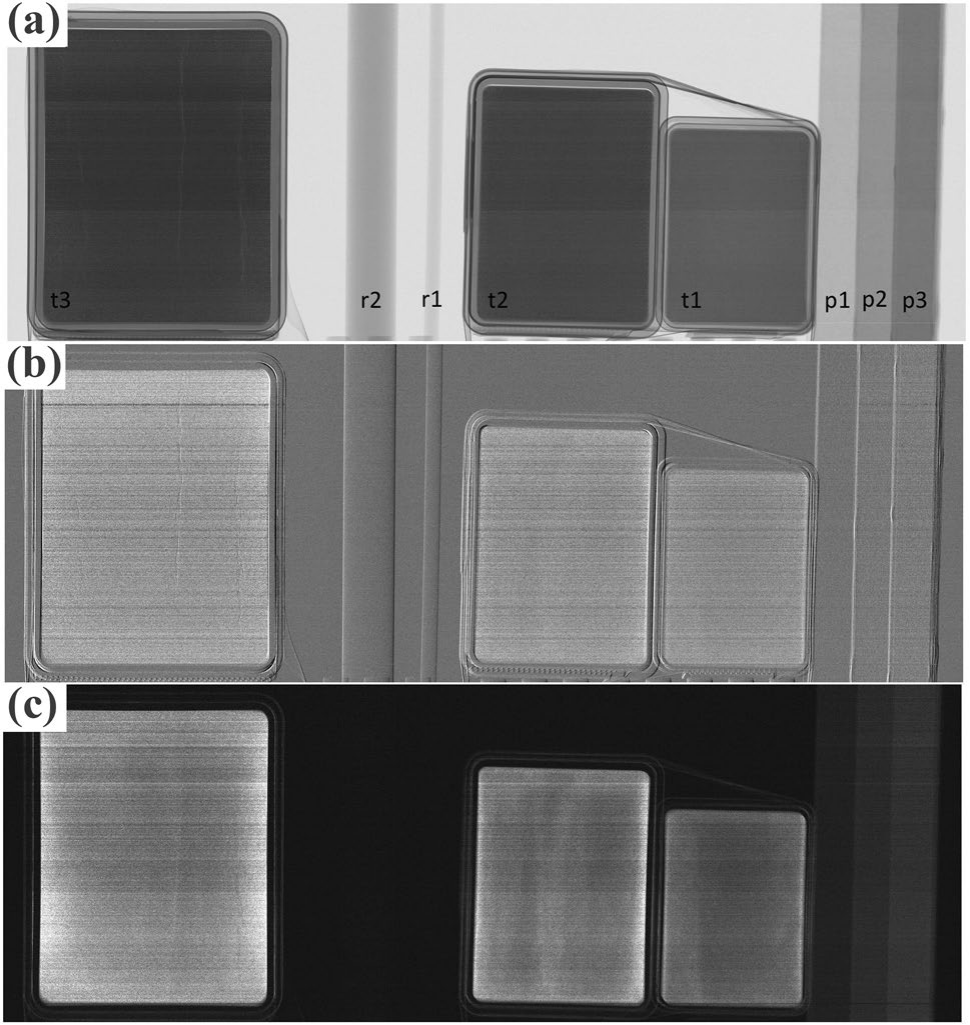
Retrieved transmission (**a**), refraction (**b**) and scatter (**c**) images of the evaluation phantom on an example image, acquired with the source-defining Huber slit (effective focal spot size) set at 70 μm. The different phantom components are labelled in panel (a) as: t1–3: boxes containing talcum powder with thickness 12 mm, 18 mm and 24 mm, respectively; r1–2: PMMA rods with diameter 3 mm and 10 mm, respectively; p1–3: stacks of paper with thickness 10 mm, 20 mm and 30 mm, respectively.

Multi-modal images were obtained by scanning a range of attenuating, refracting and scattering samples (chalk powder and paper stacks in different thicknesses and PMMA rods) through the system with a speed of 0.75 mm/s. The detector threshold was set at approximately 25 keV, however this is subject to fluctuations across the sixteen 128 × 128 pixel modules that make up our 128 × 2024 pixel strip, which explains the slight “banding” visible in the retrieved images of Fig. 2.

The process was repeated three times while varying the Huber slit opening, i.e. the effective focal spot size, to 40, 70 and 100 μm. Examples of retrieved transmission, refraction and scatter images for the 70 μm effective source are shown in Fig. 2. As can be seen, homogeneous objects such as the PMMA rods (r1-2) disappear in the scatter image, and have a different appearance in the transmission and refraction images. Powders and paper are visible in both transmission and scatter images, but with reversed contrast, e.g. the thickest paper stack (p3) appears darker in the transmission and brighter in the scatter image because it attenuates, but also scatters, more than the background.

**Figure 3:**
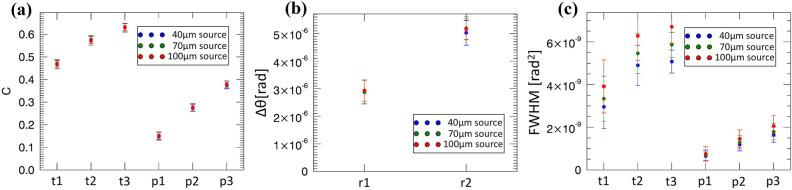
Retrieved quantitative values for attenuation (**a**), refraction (**b**) and scatter (**c**). Attenuation and scatter are reported for the three boxes containing talcum powder and for the three paper thicknesses, refraction for the two PMMA rods. Results corresponding to different (horizontal) effective focal spot sizes (summarized as “source” in the legends) are reported in different colours, namely blue, green and red for 40, 70 and 100 μm, respectively.

Quantitative values were extracted from all features, for images corresponding to all three effective source sizes. For transmission, we have selected regions of interest (ROIs) inside and outside the powder boxes and paper strips, and calculated the contrast C as [(counts outside-counts inside)/counts outside], which corresponds to the percentage attenuation of the specific detail. For refraction, we have subtracted the values of the bright and dark fringes on either side of the PMMA rods, which corresponds to the maximum span between the positive and negative refraction angles (in rad). Finally, the scatter signal corresponds to the average value inside the selected ROIs (the average of the background being zero by definition), i.e. the relative increase in variance (in rad^2^) suffered by the beamlets compared to the case where the sample is absent (see Eq. (2) in “Methods”). Results are reported in Fig. 3.

**Figure 4:**
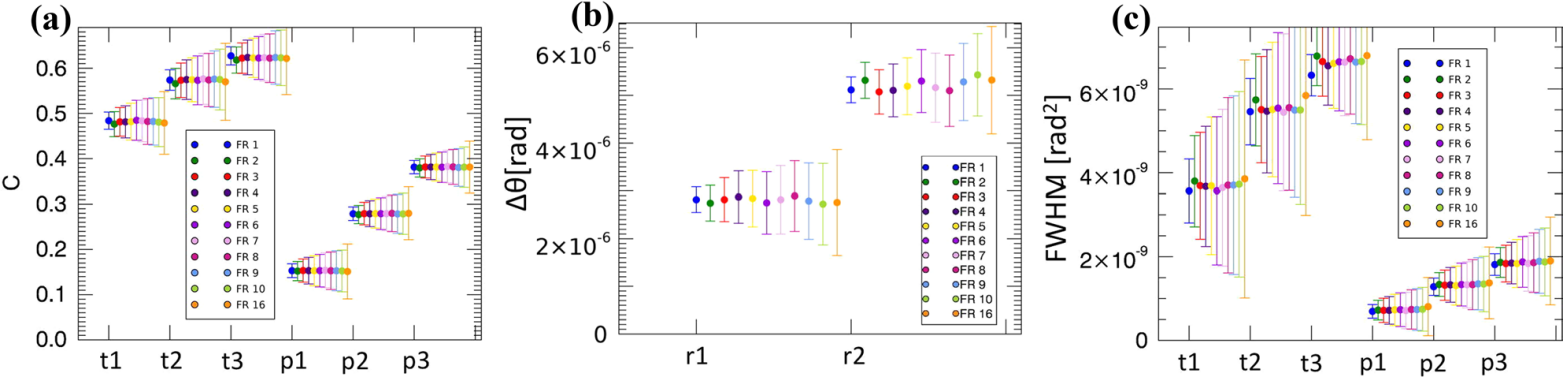
Extraction of attenuation (**a**), refraction (**b**) and scatter (**c**) values from images acquired with varying X-ray statistics, with the blue points to the far left and orange points to the far right indicating highest and lowest statistics, respectively. Used statistics ranged from 2000 to 125 detected photons per pixel at the top of the IC, which correspond to 400 and 25 photons per pixel (respectively) detected at the bottom of the IC. “FR” in the legends indicates the frame rate per second, which is the mechanism used to vary the detected statistics.

Figure 3 is the main results of this paper; features specific to each contrast channels will be discussed below. The key aspect to note is the consistency among values obtained with different effective source sizes for all contrast channels (scatter is a partial exception, also because some degree of signal saturation was observed for the thickest talcum powder boxes; this is discussed in more detail below). This is an important result, indicating that the approach provides reliable results regardless of the source size, at least up to 100 μm. For attenuation and scatter measurements, error bars were calculated from the standard deviation over the ROIs from which the signals were extracted (appropriately propagated in the case of attenuation), as this incorporates the fluctuations in the distribution of individual scatterers/attenuators in the object and therefore provides a realistic representation of the variability that can be expected in the extracted signals. For refraction, the adaptation to EI^31^ of the Modregger model based on the estimation of the standard deviation in the background of a (differential) phase retrieved image^32^ was used. We then tested the extraction of the same quantitative values against decreasing X-ray statistics. The results are shown in Fig. 4.

As can be seen, the same values are reliably extracted in all cases, with a slightly larger spread for the scatter values extracted from the thickest talcum powders, which can be partly attributed to the mentioned saturation of the signal. This takes place when the sample-induced broadening of the beamlets becomes excessive, leading to an increased overlap between them and therefore to an increased difficulty in estimating their width (see retrieval equations in the methods section). Some evidence of this is presented below (Fig. 7). Far from saturation (e.g. for the paper layers), the extraction of scatter is extremely reliable, also with significantly reduced X-ray statistics. This as an important result in an industrial inspection context, as it indicates potential for fast scans, such as those required by in-line inspection of items transported by a conveyor belt. Signal saturation can be avoided by extending the system’s dynamic range through larger mask periods, or by employing multiple apertures to interrogate the same beamlet^33^, although part of the observed effect could be due to beam hardening effects^34^, which could not be corrected through the above measures. Increasing the beam energy would also have a similar effect, and this will be the subject of future research.

**Figure 5:**
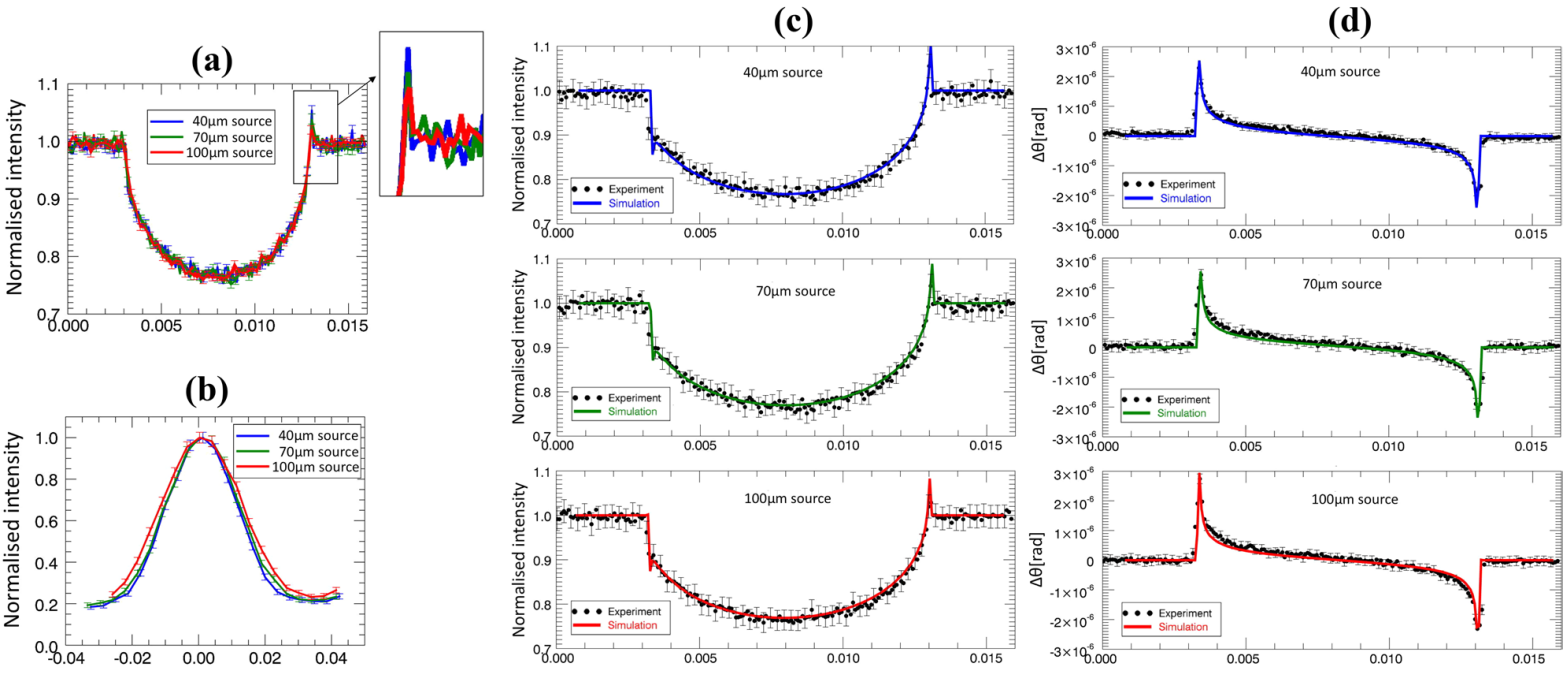
Benchmarking of refraction measurements against an established simulation method. Panel (**a**) shows the different peak height in the raw experimental profiles (acquired at approximately the same positions on the IC) corresponding to the different effective focal spot sizes (“source” in the legends); the inset shows a zoom-in of the peak region for ease of visualisation. Panel (**b**) shows the broadening of the ICs with increasing effective focal spot size. Panel (**c**) shows that the varying peak heights are correctly reproduced by the simulation when the correct focal spot size is given as input. Panel (**d**) shows that the phase retrieval process returns equivalent refraction profiles for all focal spot sizes, and that this is confirmed by the simulation.

To assess the reliability of the refraction measurements performed while varying the effective focal spot, we benchmarked them against those of an established simulation approach based on solving the Fresnel/Kirchhoff diffraction integral, which has been repeatedly validated against experimental results^5^. Results are shown in Fig. 5.

As expected, the intensity of the refraction peaks in the raw signals acquired at approximately the same IC position decreases with the increasing (effective) focal spot size, as visible in Fig. 5a. This is correctly reproduced by the simulation, which outputs predictions in agreement with the experimental results (Fig. 5c). The increased focal spot size also broadens the IC (Fig. 5b); this compensates for the peak height reduction when phase retrieval is performed, leading to the extraction of the same differential phase profiles from all plots (Fig. 5d). A broadened IC, however, has implications on the signal-to-noise ratio (SNR) of the retrieved images, i.e. on phase sensitivity, along the lines discussed in^35^. This benchmarking exercise demonstrates that the experimental refraction profiles obtained at various focal spot sizes align with the previously developed models^5,31^.

For the other two contrast channels, the data of Fig. 4 allow studying the variation of SNR with X-ray statistics. The SNR was calculated through straightforward extensions of the definitions of contrast given above, namely as [(counts outside-counts inside)/stdv(counts outside)] for attenuation, and (average value inside)/(stdev value inside) for scatter, with inside/outside referring to the selected ROI.

**Figure 6:**
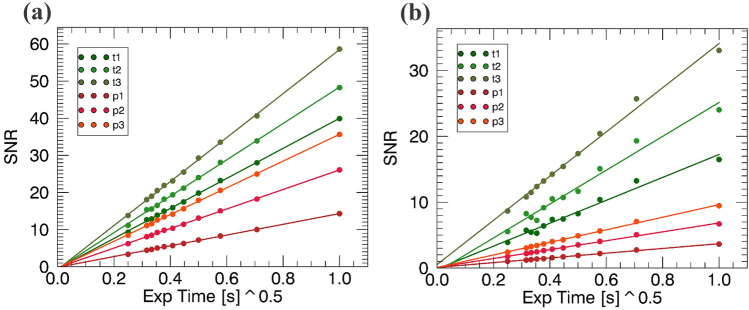
Attenuation (**a**) and scattering (**b**) SNR of the three talcum powder and paper thicknesses plotted against the detected X-ray statistics expressed in terms of the square root of the exposure time. The number of detected X-rays per pixel ranged from approximately 125/25 (top/bottom of the IC) for the lowest (0.0625 s) exposure time to approximately 2000/400 (top/bottom of the IC) for the highest (1 s). Data for slit size = 70 μm are shown.

Figure 6 shows that, for both attenuation and scatter signals, the SNR grows linearly with the square root of the exposure time, i.e. with the square root of the number of counts since measurements were carried out with the same kVp/mA source settings. This shows that our results obey Poisson statistics even after the raw images have been processed through the retrieval algorithms to isolate the attenuation and scatter signals.

**Figure 7:**
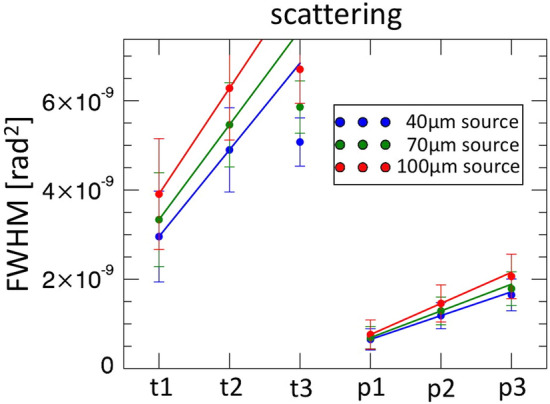
Linearity of the scatter signal against sample thickness.

Finally we evaluated the linearity of the scatter signal against sample thickness, which was demonstrated before for weak scatterers and is a pre-requisite for computed tomography implementations^33^. As can be seen in Fig. 7 the signal is linear for paper, but deviates from linearity for the (stronger scattering) talcum powder. This will require further research to determine whether linearity is re-established at higher X-ray energies leading to overall smaller scattering angles, and whether measures can be adopted to extend the linearity range by increasing the dynamic range of the system by increasing the masks’ periods, or by using multiple detector apertures to analyse a single beamlet^33^. As mentioned beam hardening could also be a factor that needs examining^34^.

One additional aspect that emerges from Fig. 7 is that larger effective focal spot sizes lead to larger scatter signals; this could already be observed in Fig. 3, and does not apply to attenuation or refraction (as made clearer by the analysis of Fig. 5). Indeed scatter is inherently different from the other two contrast channels, as it is a property of both the object and the imaging system, especially its resolution^12,16,22,23,33,36,37^. However, in EI the resolution limit is determined by the size of the aperture in the pre-sample mask^38^, which here is the same for all cases. The only difference in this case is the size of the source-shaping aperture; while this broadens the IC, this should be accounted for in the retrieval process, in a similar way as was demonstrated for refraction in Fig. 5d. Like in that case, a broadened IC should lead to a comparably larger error bar, as per standard error propagation principles when the same quantity is obtained through subtraction of increasingly large quantities with the same difference. However, in this case a larger retrieved scattering value is systematically observed for all samples and in all conditions.

**Figure 8:**
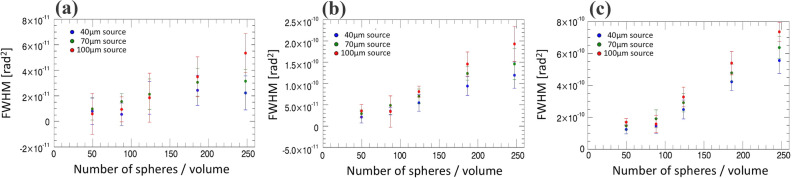
Scatter signals retrieved from simulated images of increasing concentrations of monodisperse microspheres with radius 0.75 μm (**a**), 1.5 μm (**b**) and 3 μm (**c**). Three different focal spot sizes (40, 70 and 100 μm) were used in all cases, as indicated in the graph legends.

In an attempt to reproduce this effect, we used an experimentally validated Monte-Carlo simulation^39^, by simulating increasing concentrations of microspheres, imaging them with a simulated replica of our experimental system while varying the focal spot size to 40, 70 and 100 μm, and retrieving the scatter signals using the same algorithm applied to the experimental data. Briefly, the simulation is based on the inclusion of EI masks in the McXtrace engine described in reference^40^. These are user-defined, and in this case they were matched to the parameters of the experimentally used masks (see methods section). Data are provided in input to describe the source spectrum, material refractive index and geometry, details on which are provided in the methods section. Results are shown in Fig. 8.

**Figure 9:**
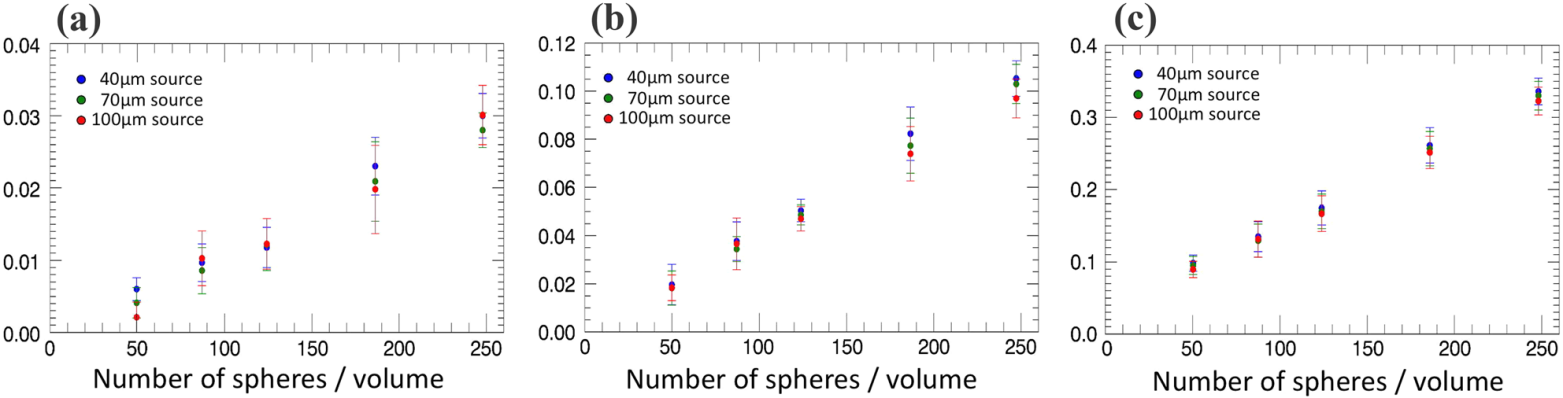
Attenuation signals retrieved from the same set of simulated images used to extract the scatter signals shown in Fig. 8 (increasing concentrations of monodisperse microspheres with radius 0.75 μm (**a**), 1.5 μm (**b**) and 3 μm (**c**)).

We explored a wide (but sub-saturation) range of scattering signals by using the same microsphere concentrations but varying their radii (0.75, 1.5 and 3.0 μm), with larger spheres leading to stronger signals. All radii were smaller than the 21 μm apertures in the pre-sample mask, which determine the ultimate resolution achievable with our system^38^. As can be seen from the figure, the experimentally observed behaviour is reproduced in most cases, with larger scatter signals typically being observed for larger focal spots. Additional research is required to clarify the origins of this effect, however the fact that it was consistently observed through repeated, independent experimental measurements, and confirmed by an experimentally validated Monte Carlo simulation, suggests it may arise from a real physical phenomenon. As a final check, we also extracted attenuation values from all the simulated microsphere images, with results reported in Fig. 9. As can be seen, in this case values are randomly ordered, with no apparent correlation to the focal spot size.

For both Figs. 8 and 9, error bars were obtained as the standard deviation over the ROI from which the signals were extracted, as previously done for the experimental results. Since the simulated microspheres were randomly distributed, these include the effect of the variability in the distribution of the scattering/attenuating centres, thus reflecting the variability that can be expected in the extracted signals.

## Conclusions

This paper demonstrates that a high-energy, scanning based multi-modal X-ray imaging system aimed at industrial inspection can be used reliably with a variety of focal spot sizes, which makes its deployment more flexible and cost-effective. We showed that the same values for the various signals are consistently extracted for all the used (effective) focal spot sizes, with the exception of scatter where the signal was observed to grow slightly with the focal spot. This is an unexpected result requiring further research, for which here we provide initial support through an experimentally validated Monte Carlo simulation. Away from saturation the scatter signal is linear against detail thickness, which makes the technology suitable for CT implementations. Saturation of the scatter signal can be avoided by extending the system’s dynamic range through larger mask periods, or by employing multiple apertures to interrogate the same beamlet^33^; increasing the X-ray energy is expected to have a similar effect, and this will be explored in a follow-on study. The SNR of the attenuation and scatter channels was shown to follow Poisson behaviour, even after the application of the retrieval algorithms to the raw input images. We find these results supportive for the use of multi-modal X-ray imaging in industrial inspections and beyond. A key limitation of this study is the relatively narrow range of explored focal spot sizes, with the lower and upper bounds based on the necessity to produce a sufficient flux for inspection applications and on previous indications of a significant reduction in phase sensitivity^30,31^, respectively. The findings reported in this paper provide a first indication on the potential use of focal spots larger than 100 μm at least for scatter measurements, which will be further explored in future research.

## Methods

The imaging system uses a COMET MXR-160HP/11 X-ray source (Comet, Wünnewil-Flamatt, Switzerland), operated at 90 kVp and 7.7 mA. It has two operation modes, 1.8 kW and 0.8 kW with 1 mm and 0.4 mm focal spots, respectively. We used the latter as it leads to a higher flux density when bracketed with a slit restricting the emission area to 40, 70 and 100 μm (along one direction). A tuneable Huber (Huber Diffraktionstechnik GmbH & Co. KG, Rimsting, Germany) slit was placed against the source output window. The targeted effective focal spot values were set manually, then measured aiming for a ± 2 μm tolerance: the slit aperture was adjusted and the measurement repeated until the 2 μm target was met. The source was uncollimated in the vertical direction. “Horizontal” and “vertical” refer to the system orientation seen in Fig. 1c; the horizontal slit opening is seen from the top in Fig. 1a, b, where the “vertical” direction enters the plane of the drawing.

The detector is a single photon counting Cd-Te CMOS (XCounter XC-FLITE FX2, Direct Conversion, Danderyd, Sweden) with 2048 × 128 square pixels 100 μm in side, placed at approximately 2.1 m from the source. Pre-sample and detector masks were placed at 1.60 m and 2.06 m from the source, respectively. The latter was 20 cm tall and featured 128 × 28 μm wide apertures (one per detector pixel), with a regular period of 98 μm. The former was 15 cm tall and featured 128 × 21 μm wide apertures arranged in a 5-way asymmetric pattern^24^, where a regular 75 μm period was sequentially altered by − 20.8 μm, − 10.4 μm, 0 μm, 10.4 μm and 20.8 μm. Both masks were fabricated by Microworks GmbH (Karlsruhe, Germany) by electroplating approximately 300 μm of gold on a 1 mm thick graphite substrate. Both masks are mounted on pairs of linear translators for movement along and across the optical axis (Newport, Irvine, CA), and on a cradle for rotation around it (Kohzu, Kawasaki, Japan).

An additional Newport translator is used to scan the samples across the field of view (as per Fig. 1a) with speed ranging from 0.75 mm/s (high statistics) to 12 mm/s (low statistics). Detector lines corresponding to the same “misaligned” pre-sample aperture are summed to form 5 images with differing illumination levels, which are fed to the retrieval algorithm to output transmission, refraction and scatter images.

The following five-Gaussian function is used to fit the IC values of five adjacent pixels:

1 $$f_{IC} \left( x \right) = \mathop \sum \limits_{i = 1}^{5} \frac{{a_{0i} }}{{\sqrt {2\pi } a_{2i} }}e^{{ - \frac{1}{2}\frac{{\left( {x - a_{1i} } \right)^{2} }}{{a_{2i}^{2} }}}} + a_{3}$$

where *x* is the pre-sample mask lateral position, and *i* is the pixel index. The fitting parameters *a*_*0i*_, *a*_*1i*_, and *a*_*2i*_ are the amplitude, shift and width of the i-th gaussian, respectively, while a_3_ is the function offset, representing the X-ray transmission through the mask septa. The sample properties are measured on a pixel-by-pixel basis as modifications of the IC curve, based on following approach:

2 $$f_{sample} \left( x \right) = \mathop \sum \limits_{i = 1}^{5} \frac{{a_{0i} s_{0} }}{{\sqrt {2\pi \left( {a_{2i}^{2} + s_{2}^{2} } \right)} }}e^{{ - \frac{1}{2}\frac{{\left( {x - a_{1i} - s_{1} } \right)^{2} }}{{a_{2i}^{2} + s_{2}^{2} }}}} + a_{3} s_{3}$$

where s_0_, s_1_, and s_2_ represent the sample’s transmission, refraction and scattering, respectively, and s_3_ takes in account any possible modulation in the IC offset caused by the sample.

To compare refraction results against theoretical predictions, a wave-optics model was used, which calculates image profiles by numerically solving the Fresnel/Kirchhoff diffraction integral, and takes into account the effects of finite source size and detector resolution through subsequent convolutions^5^. Reproducing scatter images requires generating multiple scattering events from randomly distributed centres that are smaller than the system’s resolution. For this, we used a Monte-Carlo based approach that includes refraction (McXtrace,^40^), which had been previously adapted to EI^39^. This model enables the inclusion of sample and detector masks with parameters matching the ones used experimentally and placed at the same distances (see above), and receives in input a spectral distribution (generated with TASMIC^41^ and matching the 90 kVp voltage used experimentally) and a focal spot distribution (square and equal to the three slit sizes). It also allows the generation of a random distribution of microspheres as samples, with radii 0.75, 1.5 and 3.0 μm and spectrally weighted average *β* and *δ* values equal to 6.2 × 10^–10^* and *5.9 × 10^–7^, respectively. We note that, in the approximation of fully absorbing masks (which is reasonable in this case as the transmission of a 90 kVp tungsten spectrum through 300 μm of gold is lower than 3%), the EI method is fully achromatic^42^, hence the simulation results can be assumed to be independent from the specific spectral shape.

Two PMMA rods (diameters 3 and 10 mm), three stacks with a different number of paper sheets (thicknesses 10, 20 and 30 mm) and three boxes containing talcum powder (thicknesses 12, 18 and 24 mm) were used as samples. The PMMA rods were used to measure refraction. Paper and talcum are known to produce a noticeable scatter signal, while simultaneously allowing the extraction of transmission values.
